# Montreal Cognitive Assessment vs the Mini-Mental State Examination as a Screening Tool for Patients With Genetic Frontotemporal Dementia

**DOI:** 10.1212/WNL.0000000000213401

**Published:** 2025-02-14

**Authors:** Liset de Boer, Jackie M. Poos, Esther Van Den Berg, Julie F.H. De Houwer, Tine Swartenbroekx, Elise G.P. Dopper, Pam Boesjes, Najlae Tahboun, Arabella Bouzigues, Phoebe H. Foster, Eve Ferry-Bolder, Kerala Adams-Carr, Lucy L. Russell, Rhian S. Convery, Jonathan D. Rohrer, Harro Seelaar, Lize C. Jiskoot

**Affiliations:** 1 Department of Neurology and Alzheimer Center Erasmus MC, Erasmus MC University Medical Centre, Rotterdam, the Netherlands; and; 2 Dementia Research Centre, University College London, United Kingdom.

## Abstract

**Background and Objectives:**

With upcoming clinical trials targeting preclinical stages of genetic frontotemporal dementia (FTD), early detection through cognitive screening is crucial. The Mini-Mental State Examination (MMSE) and Montreal Cognitive Assessment (MoCA) have potential as screening instruments for early-stage genetic FTD. However, no comparative evaluation has been performed. We aimed to compare MMSE and MoCA performance among presymptomatic, prodromal, and symptomatic pathogenic variant carriers to analyze which screening test has superior discriminative abilities.

**Methods:**

We used cross-sectional and longitudinal data from 2 longitudinal genetic FTD cohort studies in the Netherlands and the United Kingdom, collected between 2021 and 2024. Participants were either presymptomatic, prodromal, or symptomatic pathogenic variant carriers or healthy controls (first-degree family members without pathogenic variants for FTD). Grouping was based on the global CDR-plus-NACC-FTLD score. Participants were assessed with both MoCA and MMSE. Statistical analyses compared total and subscores between groups and evaluated predictive and classification accuracy of both tests.

**Results:**

A total of 243 participants (mean age 49.9 ± 13.1 years, mean education 14.5 ± 3.0 years, 56% female), 157 of whom were pathogenic variant carriers (*MAPT*, *GRN*, *C9orf72*, *TARDBP*, and *TBK1*) and 86 controls, were included. Carriers were classified as presymptomatic (n = 119), prodromal (n = 18), or symptomatic (n = 20). Both MoCA [*F*(3,239) = 16.565, *p* < 0.001] and MMSE [*F*(3,239) = 13.529, *p* < 0.001] total scores differed significantly between groups, with controls (median MoCA 28.5, 95% CI 28.0–29.0; median MMSE 30, 95% CI 30.0–30.0) outperforming prodromal (median MoCA 26, 95% CI 23.0–27.0; median MMSE 29, 95% CI 27.5–29.5) and symptomatic (median MoCA 20.5, 95% CI 17.0–24.0; median MMSE 26, 95% CI 23.5–29.0) carriers. MoCA distinguished between presymptomatic carriers and controls (median MoCA 28, 95% CI 27.0–29.0), but MMSE did not. MoCA demonstrated superior discriminative ability compared with MMSE (MoCA area under the curve [AUC] = 0.87, 95% CI 0.81–0.94; MMSE AUC = 0.80, 95% CI 0.72–0.89).

**Discussion:**

Its higher sensitivity and better discriminative power make MoCA a more valuable tool for cognitive screening in upcoming clinical trials targeting preclinical FTD. Future studies should aim for larger sample sizes from additional study centers.

## Introduction

Frontotemporal dementia (FTD) is a clinically, pathologically, and genetically heterogeneous type of early-onset dementia, characterized by atrophy of primarily the frontal and/or temporal lobes.^1^ The clinical profile shows behavioral, language, and/or motor disturbances with cognitive deficits in executive function and social cognition, while memory and visuospatial abilities are relatively spared.^2^ In up to 40% of patients, an autosomal dominant pattern of inheritance is found. Pathogenic variants in the progranulin (*GRN*) and microtubule-associated protein tau (*MAPT*) gene and pathogenic G4C2 repeat expansions in chromosome 9 open reading frame 72 (*C9orf72*) are the most common causes.^3^ With promising avenues opening for clinical trials, research in the genetic FTD field has been increasingly moving toward the presymptomatic and early prodromal stages, in which the pathologic burden is still low.^4^ This highlights the importance of accurate cognitive screening in these early disease stages.

The Mini-Mental State Examination (MMSE)^5^ is one of the most widely used cognitive screening instruments for dementia. However, one of the main points of critique is the lack of tasks for executive function and the low complexity of the language tasks, which can be problematic when there are isolated problems in these 2 domains, for example, in early disease stages of FTD (often rendering MMSE performance normal).^6^ The newer Montreal Cognitive Assessment (MoCA)^7^ was developed to overcome the limitations of the MMSE by being able to detect more subtle cognitive deficits, as it comprises a more comprehensive assessment of the major cognitive domains, especially executive function and language, but also short-term memory and visuospatial processing.^8^ For any given MoCA score in dementia syndromes associated with a dysexecutive syndrome, including FTD, a trend toward a higher MMSE score was found.^9^ When comparing the psychometric properties and diagnostic accuracy of the MoCA and MMSE in patients with behavioral variant FTD (bvFTD), the MoCA demonstrated high diagnostic accuracy (i.e., area under the curve, AUC = 0.93), and using a cutoff of 17/30 proved to be superior to the MMSE in terms of classification accuracy.^6^ The MoCA demonstrated high diagnostic accuracy (i.e., area under the curve, AUC = 0.93), and using a cutoff of 17/30 proved to be superior to the MMSE in terms of classification accuracy. Despite its potential as a sensitive screening instrument for the early stages of FTD, no comparative evaluation of the MoCA and MMSE has been performed in presymptomatic or prodromal pathogenic variant carriers thus far.

The aim of this study was therefore to investigate differences in test performance on the MoCA and MMSE between presymptomatic, prodromal, and symptomatic pathogenic variant carriers, in comparison with cognitively healthy controls. In addition, we explored differences between pathogenic subtypes, predictive abilities and classification accuracy, and longitudinal effects for both cognitive screening instruments.

## Methods

### Participants

We included cross-sectional data of 125 participants from the FTD Risk Cohort (FTD-RisC) of the Erasmus MC University Medical Center (Erasmus MC, Rotterdam, the Netherlands) and 118 participants from the Dementia Research Center (DRC, University College London, London, United Kingdom). Both research sites are part of the Genetic FTD Initiative (GENFI) study, in which first-degree family members of patients with FTD due to a pathogenic variant are followed on a yearly basis.^10^ Data were gathered between September 2021 and March 2024. Pathogenic variant carriers were divided into 3 groups: presymptomatic carriers, prodromal carriers, and symptomatic carriers. Carriers (n = 119) were considered presymptomatic if they did not meet the clinical diagnostic criteria for bvFTD^2^ and had a global Clinical Dementia Rating scale plus National Alzheimer's Coordinating Center Frontotemporal Lobar Degeneration Behavior and Language domains (CDR-plus-NACC-FTLD^11^) score of 0. Eighteen carriers were considered prodromal because they met the following criteria: (1) progressive deterioration of behavior, language, and/or motor functioning; (2) functional decline, evidenced by multiple study visits with global CDR-plus-NACC-FTLD^11^ = 0.5 without reversing back to 0; and (3) cognitive decline, evidenced by ≥1.5 SD below age-specific, sex-specific, and education-specific means in ≥1 domain on neuropsychological assessment. Twenty carriers were considered symptomatic because they met diagnostic criteria for definite bvFTD.^2^ Diagnoses were made in multidisciplinary consensus meetings of the respective centers, using information from Clinical Assessment (see below). DNA genotyping assigned participants to the pathogenic variant (n = 157; *C9orf72 (G4C2 expansions)* n = 79, *GRN* n = 41, *MAPT* n = 34, *TARDBP* n = 2, *TBK* n = 1) or noncarrier group (controls; n = 86). Longitudinal data were available on 97 participants (*C9orf72* n = 34, *GRN* n = 11, *MAPT* n = 21, *TARDBP* n = 2, controls n = 29).

### Standard Protocol Approvals, Registrations, and Patient Consents

This observational study was conducted in accordance with the STROBE reporting guidelines. The investigators and participants were blinded for genetic status of at-risk participants, except for those who underwent predictive testing at their own request. All participants provided written informed consent. The Erasmus MC Medical Ethics Committee gave approval for the study (MEC 2009-409). At the DRC, ethical approval for the study was gained from the local research ethics committees (NRES Committee London - Queen Square, HRA NRES Center Manchester: 140377, London - Camden & Kings Cross Research Ethics Committee: 150805).

### Clinical Assessment

All participants underwent a standardized clinical assessment, consisting of a structured interview with the participant and a knowledgeable informant (incorporating the CDR-plus-NACC-FTLD^12^), neurologic examination, neuropsychological assessment, and brain MRI. The neuropsychological assessment consisted of tests within the major cognitive domains (attention and mental processing speed, language, executive functioning, memory, visuospatial and visuoconstructive abilities, and social cognition). The MoCA^6^ and MMSE^5^ were administered as tests for global cognitive functioning. The Frontotemporal Dementia Rating Scale (FRS)^13^ was included to stage disease severity based on behavioral changes and functional decline. This caregiver questionnaire evaluates 7 aspects of a patient's personality and daily activities on a 3-point scale (never, sometimes, and all the time) reflecting the frequency of specific impairments. Scores were converted into percentages by dividing the total number of never responses by the total number of applicable questions, excluding items that were not applicable to the participant. A lower percentage indicates greater impairment of everyday abilities. The participants were grouped by the CDR-plus-NACC-FTLD; the MoCA and MMSE were not used in making this decision. The MoCA (version 8, 2018) covers 8 cognitive domains—visuospatial/executive, naming, memory, attention, language, abstract reasoning, delayed recall, and orientation—with a maximum score of 30^7^. It adds 1 point for those who have fewer than 12 years of formal education.^9^ A score of ≥26 is considered normal.^7^ The MoCA can be administered in approximately 10 minutes.^7^ The MMSE covers 6 cognitive domains—orientation, registration, attention and calculation, recall, language, and visuospatial abilities—with a maximum score of 30. A score of ≥24 is considered normal. The MMSE can be administered in 5–10 minutes.^14^

### Statistical Analysis

We conducted statistical analyses using SPSS Statistics (v28.0.1.0, IBM Corp., Armonk, NY) and created figures with R (v4.3.1, R Foundation for Statistical Computing, Vienna, Austria). Alpha was set at 0.05 across all comparisons (two-tailed), with Bonferroni corrections for multiple comparisons. Confidence intervals were provided for all parametric tests. We compared continuous demographic data between groups using one-way ANOVA for normally distributed data (with Bonferroni post hoc tests) or Kruskal-Wallis tests for non-normally distributed data (with Mann-Whitney *U* post hoc tests). Between-group differences in sex and pathogenic subtype (*MAPT*, *GRN*, *C9orf72*, *TARDBP*, and *TBK*) distribution were analyzed with Pearson χ^2^ tests. We used a general linear model with a log distribution to explore differences in MMSE and MoCA performance by study site (Rotterdam vs London) when correcting for sex, age, education, and CDR-plus-NACC-FTLD. Spearman correlations examined relationships between MoCA, MMSE, and FRS scores.

We compared performances on the MoCA and MMSE (total score and subtests) between clinical groups using nonparametric ANCOVA with age, sex, and education as covariates. We conducted an additional nonparametric ANCOVA incorporating FRS as a covariate to account for the effect of behavioral symptoms on MoCA/MMSE performance. Furthermore, we performed paired-sample t-tests to compare performances on MoCA and MMSE in every clinical group. Both ANCOVA and paired-sample *t*-test analyses were also performed in subsamples with ≤12 years (n = 84) and >12 years (n = 159) of education to explore the effects of low vs high education on MoCA/MMSE performance. We compared performances on the MoCA and MMSE between pathogenic subtypes (*C9orf72*, *GRN*, *MAPT*, *TARDBP*, and *TBK* were excluded because of a small sample size) using a nonparametric ANCOVA with age, sex, education, and CDR-plus-NACC-FTLD as covariates. To investigate whether the baseline MoCA or MMSE total scores had better predictive abilities, we built logistic regression models for each test, using group as the outcome variable, and calculated the Akaike information criterion (AIC) values for each model (AIC = 2*K – 2ln(L)). A model was considered significantly better when it was more than 2 AIC units lower than the other model. We determined sensitivity and specificity by area under the curve (AUC) by receiver-operating characteristics (ROC) analyses to investigate the classification abilities of the MoCA and MSE. An AUC >0.80 was considered to have excellent discrimination abilities.^15^ Cutoff levels for the total group and the subgroups with low and high education were given by the highest Youden Index.^16^ Finally, we conducted a generalized linear mixed model to evaluate decline (based on CDR-plus-NACC-FTLD global score) over time of the MoCA and MMSE, including age, education, and sex as covariates, with repeated measures on 2 time points.

### Data Availability

Data not provided in the article may be shared (anonymized) at the request of a qualified investigator for purposes of replicating procedures and results.

## Results

### Demographics and Clinical Data

Demographic and clinical data are listed in Table 1. There were no differences between presymptomatic, prodromal, and symptomatic carriers in sex distribution [χ^2^(3) = 4.671, *p* = 0.198, 95% CI 0.186–0.202]. Symptomatic carriers had fewer years of education than presymptomatic carriers and controls [H(3) = 11.153, *p* = 0.011]. In addition, symptomatic carriers were older than both presymptomatic carriers [*F*(3,239) = 16.236, 95% CI 10.52–21.95, *p* < 0.001] and controls [*F*(3,239) = 10.423, 95% CI 4.55–16.29, *p* < 0.001]. Prodromal carriers were also older than presymptomatic carriers [*F*(3,239) = 14.681, 95% CI 8.70–20.66, *p* < 0.001] and controls [*F*(3,239) = 8.686, 95% CI 2.74–15.00, *p* = 0.005]. There were no significant differences between symptomatic carriers and prodromal carriers in age [*F*(3,239) = −1.555, 95% CI −6.13 to 9.24, *p* = 0.690]. Regarding differences between the 2 sites, we found significantly higher scores in the DRC (London) group on the MMSE total scores after correcting for age, sex, education, and CDR-plus-NACC-FTLD [β = −0.025, 95% CI −0.43 to 0.008, *p* = 0.005]. MoCA total scores were also higher in the DRC (London) group, but it was not significant after correcting for multiple testing [β = −0.30, 95% CI −0.060 to 0.000, *p* = 0.047]. The FRS score was significantly different in symptomatic and prodromal carriers compared with presymptomatic carriers and controls [H(3) = 45.760, *p* < 0.001). Furthermore, the FRS score correlated significantly with both MoCA (ρ = 0.330, 95% CI 0.181–0.464, *p* < 0.001) and MMSE (ρ = 0.207, 95% CI 0.050–0.354, *p =* 0.008) scores.

**Table 1:** Demographic and Clinical Data per Subgroup

	Symptomatic pathogenic variant carriers (n = 20)	Prodromal pathogenic variant carriers (n = 18)	Presymptomatic pathogenic variant carriers (n = 119)	Controls (n = 86)	Statistical differences (*p* < 0.05)
CDR-plus-NACC-FTLD	≥1	0.5	0	N/A	N/A
Subdomain scores (median, range)				N/A	N/A
Memory	1 (0–3)	0 (0–1)	0 (0–0.5)		
Orientation	0.5 (0–2)	0 (0–1)	0 (0–0.5)		
Judgment and problem solving	1 (0–2)	0 (0–0.5)	0 (0–0.5)		
Community affairs	1 (0–3)	0 (0–2)	0 (0–0.5)		
Home and hobbies	1 (0–2)	0 (0–1)	0 (0–0.5)		
Personal care	0.5 (0–3)	0 (0–1)	0 (0)		
Behavior	1 (0–2)	0 (0–0.5)	0 (0–0.5)		
Language	0.5 (0-3	0.5 (0–0.5)	0 (0–0.5)		
Age at study entry, y	61.6 ± 2.4	60.1 ± 2.6	45.4 ± 1.1	51.2 ± 1.4	Sym, pro > con > pre
Sex, male/female	11/9	4/14	52/67	40/46	n.s.
Gene	*MAPT* = 9 *GRN* = 2 *C9orf72* = 8 *TARDBP* = 1	*MAPT* = 6 *GRN* = 5 *C9orf72* = 6 *TARDBP* = 1	*MAPT* = 19 *GRN* = 34 *C9orf72* = 65 *TBK* = 1	N/A	n.s.
Clinical diagnosis	bvFTD = 20	N/A	N/A	N/A	N/A
Years of education	12.7 ± 0.6	13.5 ± 0.8	14.9 ± 0.3	14.7 ± 0.3	Sym < pre, con
Low (<12 y), n = 84	10.7 ± 1.6 (n = 12)	10.8 ± 1.7 (n = 9)	11.3 ± 1.4 (n = 36)	10.9 ± 1.7 (n = 27)	n.s.
High (>12 y), n = 159	15.6 ± 0.7 (n = 8)	16.2 ± 1.5 (n = 9)	16.5 ± 1.6 (n = 83)	16.4 ± 1.5 (n = 59)	n.s.
FRS total score	49.1 ± 7.8	82.2 ± 4.9	92.8 ± 1.6	96.5 ± 0.8	Sym = pro < pre = con
MoCA at baseline					
MoCA total [0–30]	20.5 ± 5.0	24.2 ± 4.3	27.8 ± 1.9	28.1 ± 1.7	Sym, pro < pre < con
Visuospatial/executive [0–5]	3.3 ± 1.4	3.7 ± 0.7	4.5 ± 0.7	4.6 ± 0.5	Sym < pre, con; pro < con
Naming [0–3]	2.6 ± 0.6	2.8 ± 0.7	3.0 ± 0.2	3.0 ± 0.2	Sym < pre, pro, con
Attention [0–6]	4.8 ± 1.7	5.1 ± 1.3	5.9 ± 0.5	5.9 ± 0.4	Sym, pro < pre, con
Language [0–3]	1.5 ± 1.2	2.1 ± 0.9	2.7 ± 0.7	2.8 ± 0.5	Sym, pro < pre, con
Abstraction [0–2]	1.1 ± 0.8	1.5 ± 0.7	1.9 ± 0.4	1.8 ± 0.4	Sym < pro, pre, con
Recall [0–5]	1.7 ± 1.8	2.9 ± 2.1	3.8 ± 1.2	4.1 ± 1.1	Sym < pro, pre, con; pre < con
Orientation [0–6]	5.5 ± 1.0	5.8 ± 0.4	6.0 ± 0.1	5.9 ± 0.3	Sym < pre
MMSE at baseline					
MMSE total [0–30]	25.7 ± 3.4	27.2 ± 4.0	29.4 ± 1.0	29.6 ± 0.7	Sym, pro < pre, con
Orientation [0–10]	8.6 ± 1.5	9.40 ± 1.2	9.9 ± 0.4	9.8 ± 0.5	Sym < pre, pro, con
Registration [0–3]	3.0 ± 0.0	2.8 ± 0.7	3.0 ± 0.0	3.0 ± 0.0	n.s.
Attention [0–5]	4.2 ± 1.6	4.1 ± 1.6	4.8 ± 0.5	4.9 ± 0.3	Pro < pre, con
Recall [0–3]	2.2 ± 1.2	2.4 ± 1.0	2.9 ± 0.5	3.0 ± 0.2	Sym < pre, con; pro < con
Language [0–8]	7.0 ± 1.4	7.6 ± 0.9	7.9 ± 0.3	8.0 ± 0.2	Sym < pre, pro, con; pro < pre, con
Construction	0.8 ± 0.4	0.9 ± 0.3	1.0 ± 0.1	1.0 ± 0.2	Sym < pre, con

Follow-up (1 y)	Symptomatic pathogenic variant carriers (n = 9)	Prodromal pathogenic variant carriers (n = 11)	Presymptomatic pathogenic variant carriers (n = 47)	Controls (n = 30)	Statistical differences (*p* < 0.05)
CDR®-plus-NACC-FTLD global score	≥1	0.5	0	N/A	
Sex, male/female	4/5	4/7	21/26	17/13	N/A
Gene	*MAPT* = 5 *GRN* = 1 *C9* = 2 *TARDBP* = 1	*MAPT* = 3 *GRN* = 2 *C9* = 5 *TARDBP* = 1	*MAPT* = 13 *GRN* = 8 *C9* = 26	N/A	N/A
Clinical diagnosis	bvFTD = 9	N/A	N/A	N/A	N/A

Abbreviations: bvFTD = behavioral-variant frontotemporal dementia; *C9orf72* = chromosome 9 open reading frame 72; CDR-plus-NACC-FTLD = Clinical Dementia Rating Scale plus National Alzheimer's Coordinating Center Frontotemporal Lobar Degeneration Behavior and Language domains; con = control; *GRN* = progranulin; *MAPT* = microtubule-associated protein tau; MMSE = Mini-Mental State Examination; MoCA = Montreal Cognitive Assessment; n.s. = not significant; N/A = not applicable; pre = presymptomatic; pro = prodromal; sym = symptomatic.

Values indicate mean ± SD.

### Group Comparison of the MoCA and MMSE Total Scores

**Figure 1:**
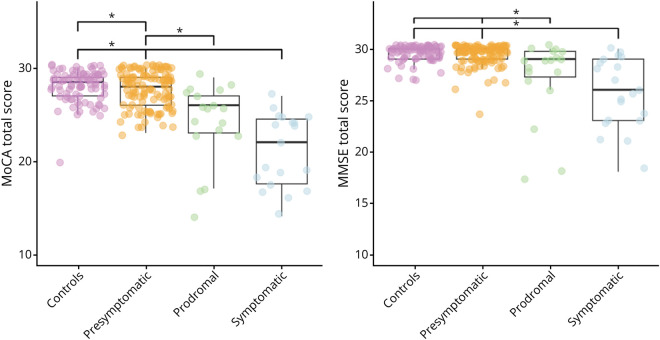
Differences in MoCA and MMSE Performance Between Clinical Groups MMSE = Mini-Mental State Examination; MoCA = Montreal Cognitive Assessment.

**Figure 2:**
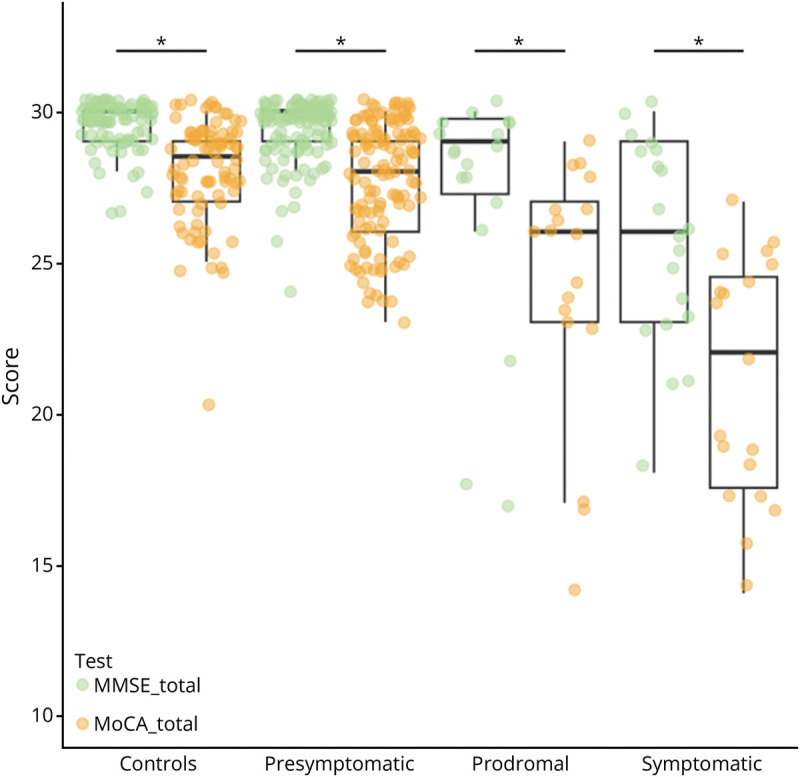
Comparison MoCA and MMSE Between Clinical Groups MMSE = Mini-Mental State Examination; MoCA = Montreal Cognitive Assessment.

Baseline MoCA total scores ranged between 7 and 30; baseline MMSE total scores ranged between 17 and 30. There were significant differences between groups in both total MoCA [*F*(3,239) = 16.565, *p* < 0.001] and MMSE [*F*(3,239) = 13.529, *p* < 0.001] scores (Figure 1). The MoCA total score was higher in controls (median MoCA 28.5, 95% CI 28.0–29.0) than in presymptomatic (median MoCA 28, 95% CI 27.0–29.0), prodromal (median MoCA 26, 95% CI 23.0–27.0), and symptomatic (median MoCA 20.5, 95% CI 17.0–24.0) carriers (*p* < 0.001–0.020). Similar patterns were found for the MMSE (median MMSE controls 30, 95% CI 30.0–30.0; prodromal 29, 95% CI 27.5–29.5; symptomatic 26, 95% CI 23.5–29.0), although the performance of presymptomatic carriers was not significantly different than of controls (*p* = 0.148). In addition, the variability in performance is higher for the MoCA compared with the MMSE in all groups. For both tests, no significant differences between prodromal and symptomatic carriers were found (*p* > 0.05). After correcting for FRS score, the statistical difference between presymptomatic carriers and controls on the MoCA and between presymptomatic and prodromal carriers on the MMSE did not remain significant. In all clinical groups, significant differences were found between MoCA and MMSE performance, with all MoCA total scores being lower than that of the MMSE (all *p* ≤ 0.001) (Figure 2). Subanalyses in participants with low (≤12 years) vs high (>12 years) education resulted in comparable findings, except for the statistical difference between presymptomatic carriers and controls not remaining significant in participants with >12 years of education (eTable 1).

### Group Comparison of the MoCA and MMSE Subscores

For the MoCA (eFigure 1), the visuospatial/executive subscore was lower in symptomatic carriers compared with presymptomatic carriers and controls [*F*(3,239) = 5.510, *p* = 0.00–0.006]. Prodromal carriers had lower scores than controls (*p* = 0.015). The attention subscore demonstrated differences in symptomatic and prodromal carriers when compared with presymptomatic carriers and controls [*F*(3,239) = 10.739, *p* < 0.001]. After FRS correction, only the difference between presymptomatic and prodromal carriers remained significant [*F*(3,158) = 2.802, *p* = 0.011]. Language performance was similarly affected, with symptomatic and prodromal carriers scoring lower than presymptomatic carriers and controls [*F*(3,239) = 12.468, *p* = 0.000–0.002]. The naming subscore also revealed differences, being lower in symptomatic carriers compared with prodromal and presymptomatic carriers and controls [*F*(3,239) = 9.222, *p* = 0.000–0.002]. After FRS correction, the difference between symptomatic and prodromal carriers did not remain significant. The abstraction subscore [*F*(3,239) = 6.483, *p* < 0.001] was lower in symptomatic carriers when compared with prodromal (*p* = 0.031) and presymptomatic carriers and controls (*p* < 0.001). These effects were not significant after correction for FRS. For the recall subscore [*F*(3,239) = 7.057], symptomatic carriers scored lower than presymptomatic carriers (*p* = 0.001) and controls (*p* < 0.001). In addition, presymptomatic carriers also scored lower on recall compared with controls (*p* = 0.042), although this difference was not significant after correction for multiple testing. After FRS correction, only the difference between symptomatic carriers and controls remained significant [*F*(3,158) = 1.698, *p* = 0.034]. Finally, the orientation subscore was lower in symptomatic carriers compared with presymptomatic carriers [*F*(3,239) = 3.576, *p* = 0.004], but these effects did not remain significant after FRS correction.

For the MMSE (eFigure 2), the orientation subscore [*F*(3,239) = 8.743] was lower in symptomatic carriers than prodromal carriers (*p* = 0.012), presymptomatic carriers (*p* < 0.001), and controls (*p* < 0.001). After FRS correction, only the difference between symptomatic carriers and presymptomatic carriers (*p* = 0.016) and controls (*p* = 0.014) remained significant [*F*(3,158) = 2.617]. No significant differences were observed in the registration subscore (*p* > 0.05). The attention subscore [*F*(3,239) = 3.420, *p* = 0.018] was lower in prodromal carriers when compared with presymptomatic carriers (*p* = 0.013) and controls (*p* = 0.002). After correcting for FRS score, these effects did not remain significant. The recall subscore [*F*(3,239) = 8.154, *p* < 0.001] was lower in symptomatic carriers than presymptomatic carriers and controls (both *p* < 0.001). Furthermore, prodromal carriers also scored lower on the recall subscore compared with controls (*p* = 0.011); however, this effect did not remain significant after correcting for FRS [*F*(3,158) = 2.752, *p* = 0.054]. In terms of language performance, this subscore [*F*(3,239) = 13.632, *p* < 0.001] was lower in symptomatic carriers compared with prodromal (*p* = 0.007) and presymptomatic carriers and controls (both *p* < 0.001). In addition, prodromal carriers had lower scores than controls (*p* = 0.012). After correcting for FRS, only the difference between symptomatic carriers and controls remained significant [*F*(3,158) = 2.429, *p* = 0.021]. The construction subscore [*F*(3,239) = 3.297, *p* = 0.021] was lower in symptomatic carriers compared with presymptomatic carriers (*p* = 0.003) and controls (*p* = 0.008).

### Differences Between Pathogenic Subtypes on MoCA and MMSE Performance

**Figure 3:**
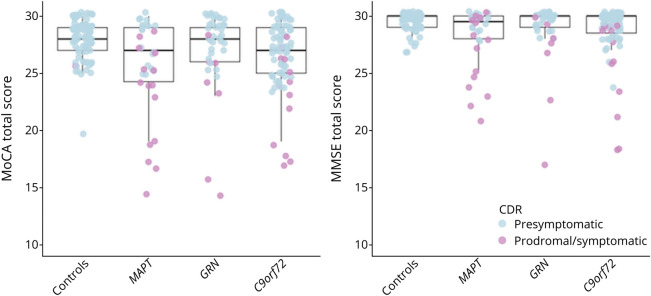
Differences in MoCA and MMSE Performance Between Genetic Groups C9orf72 = chromosome 9 open reading frame 72; CDR = CDR-plus-NACC-FTLD; GRN = progranulin; MAPT = microtubule-associated protein tau; MMSE = Mini-Mental State Examination; MoCA = Montreal Cognitive Assessment.

Only the MoCA was able to distinguish between the different pathogenic subtypes [*F*(2,151) = 4.392, *p* = 0.014] (Figure 3). After correcting for CDR-plus-NACC-FTLD, the total MoCA performance was lower in the *C9orf72* group compared with the *GRN* group (*p* = 0.004). In addition, only on the MoCA recall subscore were significant differences found [*F*(2,151) = 5.234, *p* = 0.006], with *MAPT* and *C9orf72* carriers performing lower than the *GRN* carriers.

### Predictive Abilities and Classification Accuracy of the MoCA and MMSE

The χ^2^ test for both the MoCA model (χ^2^ = 240.484, df = 15, *p* < 0.001) and MMSE model (χ^2^ = 121.936, *df* = 15, *p* < 0.001) showed a statistically significant fit. For the MoCA, the beta coefficient was exp(β) = 0.852 (95% CI 0.67–1.08) in presymptomatic carriers vs controls, exp(β) = 0.451 (95% CI 0.28–0.73) for prodromal carriers vs controls, and exp(β) = 0.386 (95% CI 0.23–0.64) for symptomatic carriers vs controls. The beta coefficient for the MMSE was exp(β) = 0.686 (95% CI 0.44–1.08) in presymptomatic carriers vs controls, exp(β) = 0.382 (95% CI 0.21–0.70) for prodromal carriers vs controls, and exp(β) = 0.369 (95% CI 0.20–0.69) for symptomatic carriers vs controls. In both models, age and FRS were significant covariates (*p* < 0.001). The AIC suggested the MoCA to be a superior model (AIC MoCA 252.484, MMSE 262.867).

**Figure 4:**
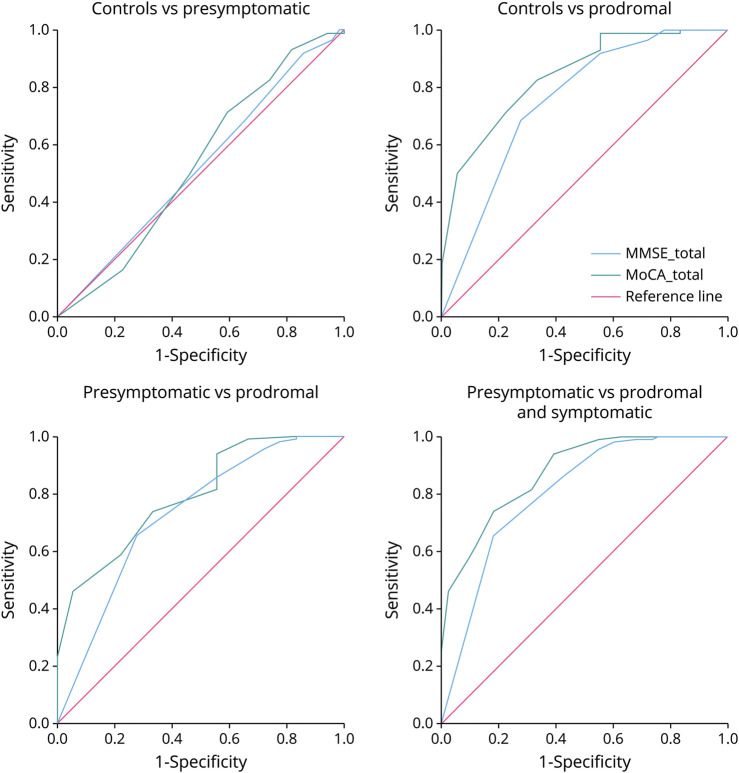
ROC Curves for Both MMSE and MoCA per Group MMSE = Mini-Mental State Examination; MoCA = Montreal Cognitive Assessment.

For distinguishing controls vs presymptomatic carriers, both MoCA (AUC = 0.534, 95% CI 0.456–0.613) and MMSE (AUC = 0.523, 95% CI 0.444–0.603) showed limited discriminative abilities. For distinguishing controls vs prodromal carriers, MoCA (AUC = 0.841, 95% CI 0.743–0.938) demonstrated better discriminative ability compared with MMSE (AUC = 0.752, 95% CI 0.613–0.897). Presymptomatic carriers vs prodromal carriers showed a similar result, although AUC values were lower (MoCA AUC = 0.793, 95% CI 0.689–0.898; MMSE AUC = 0.728, 95% CI 0.591–0.865). For distinguishing presymptomatic carriers vs prodromal carriers and symptomatic carriers, MoCA (AUC = 0.874, 95% CI 0.812–0.937) outperformed MMSE (AUC = 0.803, 95% CI 0.715–0.891), both showing an excellent discriminative ability. The ROC curve for the MoCA fully included the curve for the MMSE, indicating that there is always a cutoff for the MoCA with higher sensitivity and specificity, for any cutoff chosen for the MMSE (Figure 4). The optimal cutoff for distinguishing presymptomatic carriers from controls is 28 for the MoCA and 29 for the MMSE. For distinguishing controls and presymptomatic carriers from prodromal carriers, the optimal MoCA cutoff is 27 and the optimal MMSE cutoff is 29. For participants with **≤**12 years of education, the optimal cutoff was 26 for the MoCA and 28 for the MMSE in distinguishing controls and presymptomatic carriers, 25 and 28, respectively, for controls vs prodromal carriers, and 25 for both tests for presymptomatic carriers vs prodromal and symptomatic carriers. See Table 2 for the diagnostic classification accuracy based on the highest Youden Index and eTable 2 for AUC values and optimal cutoff scores for participants with **≤**12 years of education and participants with >12 years of education.

**Table 2:** Sensitivity and Specificity of the Cutoff Scores With the Highest Youden Index

	Cutoff	Sensitivity	Specificity	AUC
Controls vs presymptomatic				
MoCA	<28	0.71	0.41	0.53
MMSE	<29	0.92	0.14	0.52
Controls vs prodromal				
MoCA	<27	0.83	0.67	0.84
MMSE	<29	0.92	0.44	0.75
Presymptomatic vs prodromal				
MoCA	<27	0.74	0.67	0.79
MMSE	<29	0.86	0.44	0.73
Presymptomatic vs prodromal and symptomatic				
MoCA	<25	0.94	0.61	0.87
MMSE	<28	0.86	0.58	0.80

Abbreviations: AUC = area under the operating characteristic curve; MMSE = Mini-Mental State Examination; MoCA = Montreal Cognitive Assessment.

### MoCA and MMSE Performance Over Time

**Figure 5:**
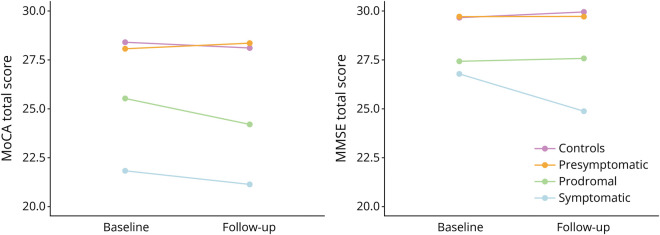
Mean Performance per Clinical Group MMSE = Mini-Mental State Examination; MoCA = Montreal Cognitive Assessment.

The effect of time was found to be statistically significant, indicating that MoCA and MMSE performance changed significantly between the 2 time points (Figure 5). The MoCA total score and time were significant predictors of the outcome variable. The coefficient was [β = −0.413, 95% CI −0.51 to −0.32, *p* < 0.001] for the MoCA total score and [β = −0.389, 95% CI −0.74 to −0.04, *p* = 0.031] for time. The same accounts for the MMSE model with total score and time coefficients of [β = −0.550, 95% CI −0.75 to −0.35, *p* < 0.001] and [β = −0.350, 95% CI −0.67 to −0.03, *p* = 0.035], respectively.

## Discussion

With promising avenues opening for clinical trials, research in the genetic FTD field has been moving toward identifying sensitive and time-effective cognitive screening instruments for the presymptomatic and early prodromal stages.^4,10^ This study compared MoCA and MMSE performance across presymptomatic, prodromal, and symptomatic carriers of pathogenic variants and healthy controls. MoCA scores were consistently lower than MMSE scores and differentiated better between presymptomatic carriers and controls. MoCA also showed greater sensitivity, especially in *C9orf72* carriers compared with *GRN* carriers, and outperformed MMSE in detecting longitudinal early cognitive decline.

Our approach contrasts with other studies in MoCA/MMSE comparisons that mostly focused solely on patients in the symptomatic phase of FTD^6,17-19^ and not on the presymptomatic and early symptomatic (prodromal) stages. The main finding from our study was that the MoCA outperformed the MMSE—in all stages of disease—but was particularly useful in identifying global cognitive decline as early as the presymptomatic stage, whereas the MMSE could not. In the GENFI study, the MMSE was significantly lower in presymptomatic carriers than in controls; however this study found that only carriers with an estimation of minus 5 years of onset had a significant lower MMSE.^10^ Our results are in line with studies comparing the MoCA and MMSE in symptomatic FTD that consistently found MoCA to be a more sensitive instrument than the MMSE.^18^ The results of this study also coincide with studies comparing the MoCA and MMSE in other patient groups. Previous studies showed that MoCA is better able to detect mild cognitive impairment (MCI), a precursor of dementia, than MMSE, with AUCs of 0.856 and 0.883 for MoCA and AUCs of 0.736 and 0.780 for MMSE, respectively.^20,21^ In patients with Parkinson disease, MoCA also outperformed MMSE, with a higher percentage of correct diagnoses.^22^ Combined, ours and previous studies suggest that the MoCA is more sensitive than the MMSE for the detection of early-stage dementia. This is most likely because the MoCA is considered more difficult than the MMSE because items measuring orientation may be easier than items assessing visuospatial and executive function.^23^ Moreover, as the MoCA additionally comprises items for executive function and language, it is presumably better able to detect dementia syndromes specifically targeting these cognitive domains, as is the case for FTD.

All MoCA/MMSE subscores, except the registration subscore of the MMSE, were significantly lower in symptomatic carriers than in presymptomatic carriers and controls. Of interest, the visuospatial/executive, attention, recall, and language subscores of the MoCA and the attention, recall, and language subtests of the MMSE were significantly lower in prodromal carriers than in presymptomatic carriers and controls. The findings are partially in line with those of a previous study that found lower visuospatial/executive, attention, language, recall, and orientation subscores in MCI due to Parkinson disease compared with healthy controls.^22^ The affected subscores coincide with the domains known to be declining in the presymptomatic stage of genetic FTD.^24^ The recall subtest of the MoCA was the only subscore that discriminated presymptomatic carriers from controls; however, correction for multiple testing rendered this finding nonsignificant. One explanation for this trend is the higher number of words (i.e., higher cognitive load) that needs to be recalled in the MoCA as compared with the MMSE.^7^ Moreover, the presymptomatic group comprises carriers with *MAPT*, *GRN*, and *C9orf72* variants that all can be accompanied by memory deficits—even as early as the presymptomatic stage.^24-26^ An interesting exploration in a future study would be to investigate how memory subscores of the MoCA are affected in these pathogenic variants in different disease stages, as previous studies have shown gene-specific memory profiles in presymptomatic and symptomatic genetic FTD.^22,24,27^

In addition, our findings demonstrate that the MoCA exhibits a stronger relationship with behavioral test scores than the MMSE, as measured by the FRS. This may be attributed to the MoCA's broad coverage of cognitive domains, such as executive functioning and attention, which are closely tied to behavioral regulation.^28,29^ When analyzing MoCA and MMSE scores across groups, we observed that the MoCA showed greater differentiation, reflecting its sensitivity to cognitive deficits closely aligned with behavioral symptomatology. In line with this hypothesis, after controlling for the FRS in our statistical analyses, the effect of the MoCA was reduced. This suggests that the MoCA captures aspects of cognitive impairment that overlap significantly with behavioral dysfunction, raising the need for caution when using these tools in predictive models or group comparisons.

The optimal cutoff scores for distinguishing groups are 28 (MoCA) and 29 (MMSE) for presymptomatic carriers vs controls and 27 (MoCA) and 29 (MMSE) for prodromal carriers vs presymptomatic carriers and controls. Although the MMSE has better sensitivity, it lacks specificity, making a cutoff of 29 impractical in clinical settings. The MoCA offers better specificity, but this specificity remains low for clinical use.^30^ Previous studies report a similar MoCA specificity in MCI and early AD.^20-22^ The cutoff scores of this study are consistent with a previous study that identified the same cutoff for the MoCA to detect FTD.^31^ In contrast, these cutoff scores are higher compared to those found in a previous study (17 and 26, respectively), potentially because of differences in education level in the two studies.^6^ Because our study included a substantial proportion of higher educated (>12 years of education, >70% of the sample) than lower educated (≤12 years of education) participants, it is possible that this has influenced our results. Indeed, when we explored these effects in a subgroup analysis in lower educated individuals, we observed lower MoCA and MMSE mean total scores and optimal cutoff scores. Because education level is one of the main indirect proxies of cognitive reserve,^32^ it is likely that higher educated participants have higher scores on the MoCA and MMSE, even in the presence of mild cognitive impairments. These findings underscore the importance of considering education level when interpreting cognitive tests because it may significantly influence its sensitivity and specificity in detecting cognitive impairments.

Furthermore, time is a significant predictor in our model, suggesting that with disease progression (i.e., higher CDR plus NACC-FTLD), both MoCA and MMSE scores decline. This trend is particularly evident in prodromal and symptomatic carriers on the MoCA and symptomatic carriers on the MMSE, showing significant decline in performance from baseline to follow-up. Our longitudinal findings are in line with a previous study that found steeper rates of decline in the MoCA compared with the MMSE in patients with early-stage bvFTD than patients with AD, potentially serving as a method for differential diagnosis.^19^

When comparing MoCA and MMSE performance across different pathogenic subtypes of FTD, our findings point in the direction of superior sensitivity of the MoCA in differentiating between these pathogenic subtypes compared with the MMSE. Specifically, overall MoCA performance was lower in the *C9orf72* carriers compared with the *GRN* group. This aligns with previous studies describing widespread impairment covering multiple cognitive domains in this FTD pathogenic variant.^24,25,27,33^ Furthermore, significant differences between pathogenic subtypes were observed for the MoCA recall subscore, with both *MAPT* and *C9orf72* carriers performing lower than *GRN* carriers. This coincides with the study by Poos et al.^24,26^ who found memory to be contributing the strongest to the gene-specific composite score in carriers with *C9orf72* and *MAPT* pathogenic variants. This is likely related to the temporal lobe atrophy, associated with memory deficits, in *C9orf72* and *MAPT* pathogenic variants.

A strength of this study is its large cohort of genetic FTD variant carriers from 2 major FTD research centers, matched with pathogenic-negative controls. This design enhances the robustness of our findings by controlling for potential genetic confounders. In addition, we classified carriers into presymptomatic, prodromal, and symptomatic stages based on CDR-plus NACC-FTLD global scores,^12^ offering a comprehensive view across the disease stages of FTD. Although genetic FTD is a rare neurodegenerative disease,^3^ one limitation lies in the small sample size, especially when stratified into different clinical groups, which might have affected the generalizability of our findings. As a result, all symptomatic carriers were pooled into one group, despite some being in the early stages (i.e., CDR-plus NACC-FTLD 1) and others being in later stages (i.e., CDR-plus NACC-FTLD >1) of disease. Moreover, we decided to pool the pathogenic subtypes (including all CDR-plus NACC-FTLD scores), which has increased statistical power, but could have influenced gene-specific analyses. Future studies should aim to recruit larger study groups, for instance, by collaborating with other study sites within the larger GENFI consortium and worldwide initiatives for genetic FTD (such as the Frontotemporal Preventive Initiative). Furthermore, given the MoCA and MMSE differences between the 2 cohorts, additional cohorts are needed to assess the generalizability of these findings across languages and cultures. Finally, investigating longitudinal data of multiple follow-ups would be recommended to delineate more accurate profiles of cognitive performance over time, aiding in early disease detection and monitoring progression in genetic FTD.

Overall, the MoCA demonstrates greater discriminative ability than the MMSE. Our findings indicate that incorporating the MoCA into routine screening assessments for individuals at risk of FTD, particularly when evaluating presymptomatic and prodromal stages, is recommendable. Future studies, including larger sample sizes and more follow-up measurements, are needed to provide better insight into the generalizability of the current findings.

## Glossary

AIC: Akaike information criterion
AUC: area under the curve
C9orf72: chromosome 9 open reading frame 72
FRS: Frontotemporal Dementia Rating Scale
FTD: frontotemporal dementia
FTD-RisC: FTD Risk Cohort
GENFI: Genetic FTD Initiative
GRN: progranulin
MAPT: microtubule-associated protein tau
MCI: mild cognitive impairment
MMSE: Mini-Mental State Examination
MoCA: Montreal Cognitive Assessment
ROC: receiver-operating characteristics
